# Methyl *N*-hy­droxy-*N*-(2-methyl­phen­yl)carbamate

**DOI:** 10.1107/S1600536813000421

**Published:** 2013-01-12

**Authors:** Binbin Zhang, Yifeng Wang, Kun Dong, Danqian Xu

**Affiliations:** a Catalytic Hydrogenation Research Center, Zhejiang University of Technology, Hangzhou 310014, People’s Republic of China

## Abstract

There are three independent mol­ecules in the asymmetric unit of the title compound, C_9_H_11_NO_3_, which are connected by O—H⋯O hydrogen bonds, forming an *R*_3_^3^(15) ring. The dihedral angles between the planes of the benzene and amide groups are 75.16 (3), 71.47 (3) and 70.56 (3)°. The hy­droxy O atom lies 0.912 (3), 1.172 (2) and 1.339 (2) Å from the mean plane of the corresponding benzene ring in the three mol­ecules.

## Related literature

The title compound is an inter­mediate in the synthesis of the strobilurin fungicide pyraclostrobin. For general background, see: Hou *et al.* (2002 ▶); Yang *et al.* (2012 ▶); Tao *et al.* (2009 ▶). For related structures, see: Mercader *et al.* (2011 ▶). For graph-set notation, see: Bernstein *et al.* (1995 ▶).
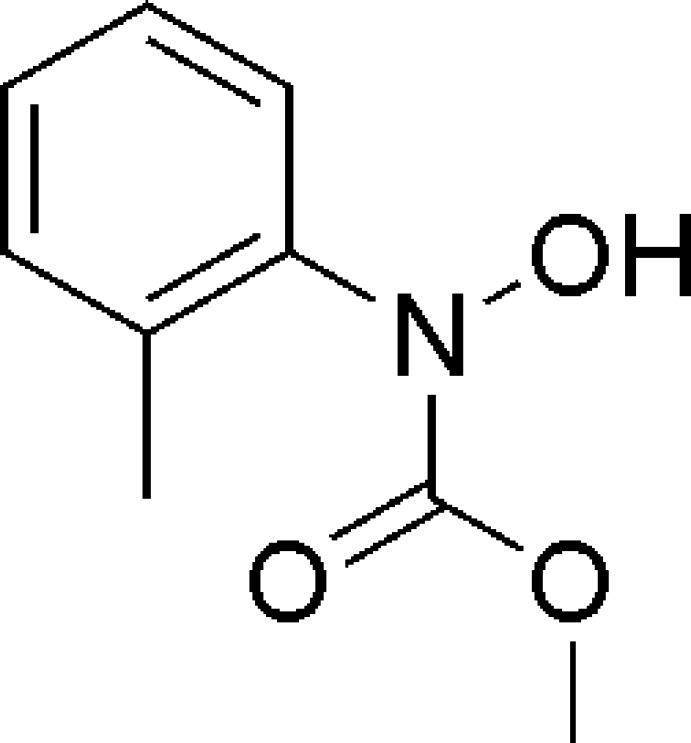


## Experimental

### 

#### Crystal data

C_9_H_11_NO_3_
*M* *_r_* = 181.19
Monoclinic, 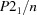

*a* = 7.6418 (3) Å
*b* = 20.8825 (9) Å
*c* = 18.0412 (9) Å
β = 94.485 (1)°
*V* = 2870.2 (2) Å^3^
*Z* = 12
Mo *K*α radiation
μ = 0.10 mm^−1^
*T* = 296 K
0.54 × 0.37 × 0.18 mm

#### Data collection

Rigaku R-AXIS RAPID/ZJUG diffractometer
Absorption correction: multi-scan (*ABSCOR*; Higashi, 1995 ▶) *T*_min_ = 0.946, *T*_max_ = 0.983
24402 measured reflections
5643 independent reflections
3163 reflections with *I* > 2σ(*I*)
*R*_int_ = 0.051

#### Refinement

*R*[*F* ^2^ > 2σ(*F* ^2^)] = 0.063
*wR*(*F* ^2^) = 0.161
*S* = 1.01
5643 reflections
362 parameters
H-atom parameters constrained
Δρ_max_ = 0.41 e Å^−3^
Δρ_min_ = −0.18 e Å^−3^

### 

Data collection: *PROCESS-AUTO* (Rigaku, 2006 ▶); cell refinement: *PROCESS-AUTO*; data reduction: *CrystalStructure* (Rigaku, 2007 ▶); program(s) used to solve structure: *SHELXS97* (Sheldrick, 2008 ▶); program(s) used to refine structure: *SHELXL97* (Sheldrick, 2008 ▶); molecular graphics: *ORTEP-3 for Windows* (Farrugia, 2012 ▶); software used to prepare material for publication: *WinGX* (Farrugia, 2012 ▶).

## Supplementary Material

Crystal structure: contains datablock(s) I, global. DOI: 10.1107/S1600536813000421/pk2462sup1.cif

Structure factors: contains datablock(s) I. DOI: 10.1107/S1600536813000421/pk2462Isup2.hkl

Supplementary material file. DOI: 10.1107/S1600536813000421/pk2462Isup3.cml

Additional supplementary materials:  crystallographic information; 3D view; checkCIF report

## Figures and Tables

**Table 1:** Hydrogen-bond geometry (Å, °)

*D*—H⋯*A*	*D*—H	H⋯*A*	*D*⋯*A*	*D*—H⋯*A*
O1*A*—H1*A*⋯O2*B*	0.82	1.94	2.719 (3)	157
O1*B*—H1*B*⋯O2*C*	0.82	1.94	2.716 (3)	157
O1*C*—H1*C*⋯O2*A*	0.82	1.99	2.757 (3)	156

**Fig. 1.**
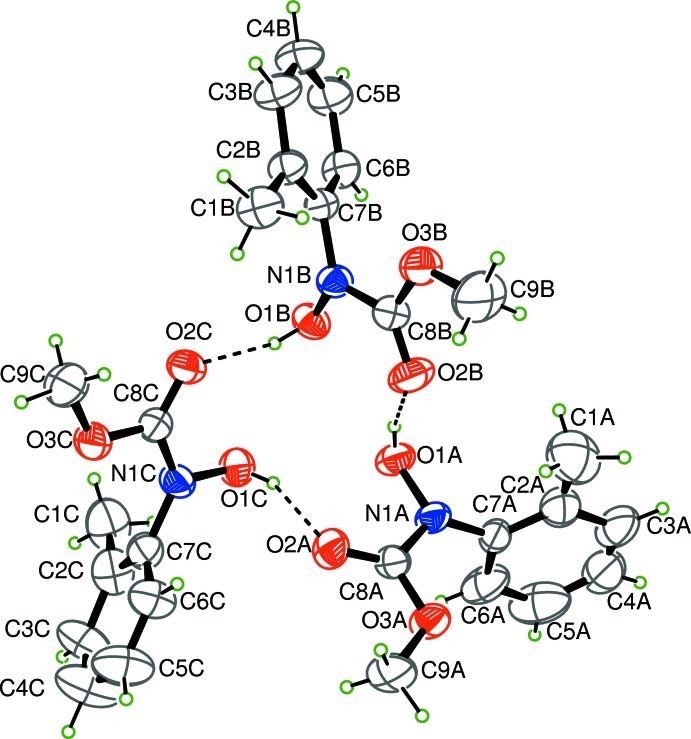
The structure of the title compound. Displacement ellipsoids are drawn at the 50% probability level.

**Fig. 2.**
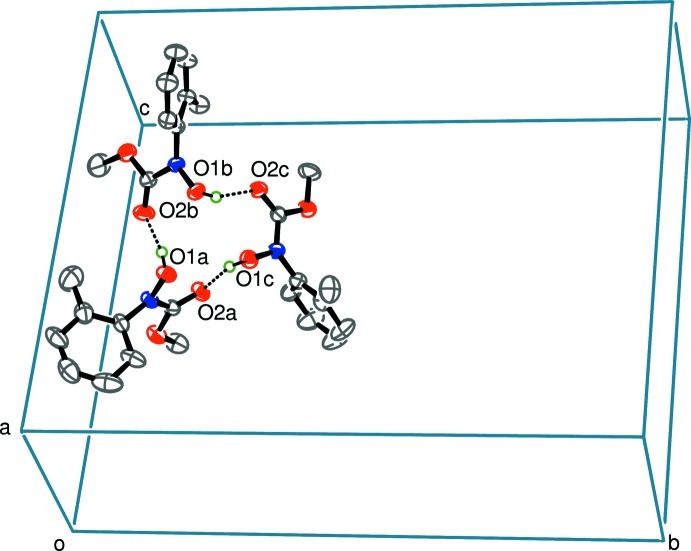
Placement of the hydrogen-bonded trimer in the unit cell.

## supplementary crystallographic information

### Comment

*N*-aryl hydroxylamines are a significant class of compounds that are key building blocks in the synthesis of natural products and biologically active compounds. The title compound, which was readily synthesized from (*N*)-(2-methylphenyl)hydroxylamine, act as an intermediate for the synthesis of Strobilurin fungicide Pyraclostrobin. In this article, the crystal structure of the title compound methyl(*N*)-hydroxy-2- methylphenylcarbamate is described (Fig. 1). There are three independent molecules in the asymmetric unit, which are connected by intermolecular O—H···O hydrogen bonds to construct a large ring involving 15 atoms with graph set notation *R*^3^_3_(15) (Fig. 2). In each molecule, the dihedral angles of the plane of the phenyl ring and the plane of the amide moiety are 75.16 (3)°, 71.47 (3)°, 70.56 (3)° respectively, while the phenyl rings of the three molecules make dihedral angles of 79.87 (3)°, 71.01 (3)°, 55.86 (3)° with each other. Each hydroxyl O atom lies 0.912 (3) Å, 1.172 (2) Å and 1.339 (2) Å from the mean plane of the corresponding phenyl ring.

### Experimental

To a solution of (*N*)-(2-methylphenyl)hydroxylamine (0.022 mol) in CH_2_Cl_2_ (20 ml), sodium bicarbonate (0.033 mol) was added and methyl chloroformate(0.024 mol) was added dropwise, and the mixture was stirred at 0° C for 2 h (monitored by HPLC). Then the reaction mixture was filtered and distilled under vacuum, and the residue was recrystallized from petroleum ether to give the title compound. Single crystals were obtained by slow evaporation of a CH_2_Cl_2_ and cyclohexane solution.

### Refinement

H atoms were placed in calculated positions with O—H = 0.82 Å, C—H = 0.96 Å (*sp*), C—H = 0.93 Å (aromatic). All H atoms included in the final cycles of refinement using a riding model, with *U*_iso_(H) = 1.2*U*_eq_ or 1.5*U*_eq_ (sp3) of the carrier atoms.

### Crystal data

**Table:**

C_9_H_11_NO_3_	*F*(000) = 1152
*M**_r_* = 181.19	*D*_x_ = 1.258 Mg m^−^^3^
Monoclinic, *P*2_1_/*n*	Mo *K*α radiation, λ = 0.71073 Å
Hall symbol: -P 2yn	Cell parameters from 13549 reflections
*a* = 7.6418 (3) Å	θ = 3.0–27.4°
*b* = 20.8825 (9) Å	µ = 0.10 mm^−^^1^
*c* = 18.0412 (9) Å	*T* = 296 K
β = 94.485 (1)°	Needle, colorless
*V* = 2870.2 (2) Å^3^	0.54 × 0.37 × 0.18 mm
*Z* = 12	

### Data collection

**Table:**

Rigaku R-AXIS RAPID/ZJUG diffractometer	5643 independent reflections
Radiation source: rotating anode	3163 reflections with *I* > 2σ(*I*)
Graphite monochromator	*R*_int_ = 0.051
Detector resolution: 10.00 pixels mm^-1^	θ_max_ = 26.0°, θ_min_ = 3.0°
ω scans	*h* = −8→9
Absorption correction: multi-scan (*ABSCOR*; Higashi, 1995)	*k* = −25→24
*T*_min_ = 0.946, *T*_max_ = 0.983	*l* = −22→22
24402 measured reflections	

### Refinement

**Table:**

Refinement on *F*^2^	Secondary atom site location: difference Fourier map
Least-squares matrix: full	Hydrogen site location: inferred from neighbouring sites
*R*[*F*^2^ > 2σ(*F*^2^)] = 0.063	H-atom parameters constrained
*wR*(*F*^2^) = 0.161	*w* = 1/[σ^2^(*F*_o_^2^) + (0.0464*P*)^2^ + 2.225*P*] where *P* = (*F*_o_^2^ + 2*F*_c_^2^)/3
*S* = 1.01	(Δ/σ)_max_ < 0.001
5643 reflections	Δρ_max_ = 0.41 e Å^−^^3^
362 parameters	Δρ_min_ = −0.18 e Å^−^^3^
0 restraints	Extinction correction: *SHELXL97* (Sheldrick, 2008), Fc^*^=kFc[1+0.001xFc^2^λ^3^/sin(2θ)]^-1/4^
Primary atom site location: structure-invariant direct methods	Extinction coefficient: 0.0071 (7)

### Special details

**Table:**

Geometry. All e.s.d.'s (except the e.s.d. in the dihedral angle between two l.s. planes) are estimated using the full covariance matrix. The cell e.s.d.'s are taken into account individually in the estimation of e.s.d.'s in distances, angles and torsion angles; correlations between e.s.d.'s in cell parameters are only used when they are defined by crystal symmetry. An approximate (isotropic) treatment of cell e.s.d.'s is used for estimating e.s.d.'s involving l.s. planes.
Refinement. Refinement of *F*^2^ against ALL reflections. The weighted *R*-factor *wR* and goodness of fit *S* are based on *F*^2^, conventional *R*-factors *R* are based on *F*, with *F* set to zero for negative *F*^2^. The threshold expression of *F*^2^ > σ(*F*^2^) is used only for calculating *R*-factors(gt) *etc*. and is not relevant to the choice of reflections for refinement. *R*-factors based on *F*^2^ are statistically about twice as large as those based on *F*, and *R*- factors based on ALL data will be even larger.

### Fractional atomic coordinates and isotropic or equivalent isotropic displacement parameters (Å^2^)

**Table:**

	*x*	*y*	*z*	*U*_iso_*/*U*_eq_	
C1A	0.7015 (7)	0.0013 (2)	0.4220 (3)	0.1281 (17)	
H1A1	0.8160	0.0147	0.4417	0.192*	
H1A2	0.6990	−0.0445	0.4173	0.192*	
H1A3	0.6157	0.0146	0.4550	0.192*	
C2A	0.6622 (5)	0.02987 (19)	0.3507 (3)	0.0855 (11)	
C3A	0.6870 (5)	−0.0069 (3)	0.2864 (3)	0.1027 (15)	
H3A	0.7291	−0.0486	0.2901	0.123*	
C4A	0.6481 (5)	0.0204 (3)	0.2205 (4)	0.1099 (17)	
H4A	0.6686	−0.0035	0.1785	0.132*	
C5A	0.5789 (6)	0.0819 (3)	0.2091 (2)	0.1206 (18)	
H5A	0.5485	0.0977	0.1616	0.145*	
C6A	0.5584 (5)	0.1173 (2)	0.2709 (2)	0.0930 (13)	
H6A	0.5189	0.1593	0.2662	0.112*	
C7A	0.5973 (3)	0.09029 (14)	0.34306 (18)	0.0586 (8)	
C8A	0.4200 (4)	0.15525 (14)	0.42474 (15)	0.0522 (7)	
C9A	0.1141 (4)	0.1418 (2)	0.4157 (2)	0.0876 (12)	
H9A1	0.1087	0.1362	0.4683	0.131*	
H9A2	0.0244	0.1164	0.3896	0.131*	
H9A3	0.0962	0.1861	0.4032	0.131*	
N1A	0.5733 (3)	0.13103 (12)	0.40482 (13)	0.0576 (6)	
O1A	0.7214 (2)	0.16837 (11)	0.42788 (11)	0.0633 (6)	
H1A	0.7555	0.1589	0.4707	0.095*	
O2A	0.4081 (3)	0.20012 (10)	0.46662 (12)	0.0667 (6)	
O3A	0.2843 (2)	0.12164 (11)	0.39471 (12)	0.0685 (6)	
C1B	0.9234 (4)	0.19851 (18)	0.78477 (18)	0.0734 (9)	
H1B1	0.8450	0.2246	0.7535	0.110*	
H1B2	0.9386	0.2171	0.8335	0.110*	
H1B3	0.8751	0.1563	0.7881	0.110*	
C2B	1.0991 (4)	0.19463 (14)	0.75196 (16)	0.0552 (7)	
C3B	1.2551 (4)	0.19938 (17)	0.79671 (18)	0.0725 (9)	
H3B	1.2507	0.2035	0.8479	0.087*	
C4B	1.4155 (4)	0.19809 (18)	0.7672 (2)	0.0772 (10)	
H4B	1.5179	0.2008	0.7984	0.093*	
C5B	1.4247 (4)	0.19279 (17)	0.6920 (2)	0.0735 (9)	
H5B	1.5333	0.1930	0.6721	0.088*	
C6B	1.2733 (4)	0.18723 (14)	0.64585 (17)	0.0593 (8)	
H6B	1.2791	0.1835	0.5947	0.071*	
C7B	1.1122 (3)	0.18725 (13)	0.67609 (15)	0.0478 (6)	
C8B	0.8634 (4)	0.12549 (15)	0.61676 (18)	0.0586 (7)	
C9B	0.7983 (6)	0.02666 (19)	0.6715 (3)	0.1248 (18)	
H9B1	0.6769	0.0378	0.6732	0.187*	
H9B2	0.8351	0.0009	0.7139	0.187*	
H9B3	0.8136	0.0030	0.6268	0.187*	
N1B	0.9562 (3)	0.18018 (11)	0.62738 (12)	0.0525 (6)	
O1B	0.9443 (3)	0.22088 (9)	0.56502 (10)	0.0586 (5)	
H1B	0.8743	0.2497	0.5714	0.088*	
O2B	0.7543 (3)	0.11544 (11)	0.56597 (13)	0.0803 (7)	
O3B	0.9030 (3)	0.08435 (10)	0.67221 (13)	0.0744 (6)	
C1C	0.5743 (5)	0.43964 (19)	0.4292 (3)	0.1070 (14)	
H1C1	0.6516	0.4438	0.4736	0.161*	
H1C2	0.5595	0.4807	0.4056	0.161*	
H1C3	0.6238	0.4101	0.3958	0.161*	
C2C	0.4036 (5)	0.41579 (18)	0.4486 (2)	0.0846 (11)	
C3C	0.2437 (6)	0.4432 (2)	0.4177 (2)	0.1088 (15)	
H3C	0.2472	0.4771	0.3844	0.131*	
C4C	0.0889 (7)	0.4210 (3)	0.4356 (3)	0.1309 (19)	
H4C	−0.0135	0.4403	0.4152	0.157*	
C5C	0.0768 (5)	0.3698 (3)	0.4841 (3)	0.1148 (16)	
H5C	−0.0324	0.3549	0.4956	0.138*	
C6C	0.2236 (4)	0.34211 (18)	0.5139 (2)	0.0800 (10)	
H6C	0.2175	0.3073	0.5457	0.096*	
C9C	0.5530 (5)	0.3753 (2)	0.71892 (19)	0.0984 (13)	
H9C1	0.5448	0.3326	0.7379	0.148*	
H9C2	0.4735	0.4027	0.7427	0.148*	
H9C3	0.6708	0.3908	0.7288	0.148*	
C7C	0.3899 (4)	0.36664 (15)	0.49641 (17)	0.0646 (8)	
C8C	0.6045 (4)	0.33602 (14)	0.60086 (17)	0.0547 (7)	
N1C	0.5431 (3)	0.33508 (12)	0.52944 (13)	0.0592 (6)	
O1C	0.6448 (3)	0.29974 (10)	0.48250 (11)	0.0627 (6)	
H1C	0.6012	0.2642	0.4754	0.094*	
O2C	0.7308 (3)	0.30629 (11)	0.62704 (11)	0.0682 (6)	
O3C	0.5079 (3)	0.37516 (11)	0.64001 (12)	0.0741 (6)	

### Atomic displacement parameters (Å^2^)

**Table:**

	*U*^11^	*U*^22^	*U*^33^	*U*^12^	*U*^13^	*U*^23^
C1A	0.134 (4)	0.114 (4)	0.136 (4)	0.002 (3)	0.007 (3)	0.027 (3)
C2A	0.063 (2)	0.081 (3)	0.112 (3)	−0.0024 (19)	0.002 (2)	−0.017 (2)
C3A	0.072 (3)	0.126 (4)	0.109 (4)	0.005 (2)	0.004 (2)	−0.063 (3)
C4A	0.065 (3)	0.123 (4)	0.143 (5)	0.003 (3)	0.014 (3)	−0.062 (4)
C5A	0.090 (3)	0.206 (6)	0.066 (3)	−0.009 (4)	0.006 (2)	−0.016 (3)
C6A	0.079 (2)	0.137 (4)	0.063 (2)	−0.012 (2)	0.0030 (19)	−0.037 (2)
C7A	0.0411 (15)	0.0571 (18)	0.078 (2)	−0.0017 (14)	0.0078 (14)	−0.0214 (16)
C8A	0.0461 (16)	0.0638 (18)	0.0470 (16)	0.0028 (14)	0.0054 (13)	−0.0060 (14)
C9A	0.0410 (17)	0.133 (3)	0.091 (3)	0.0032 (19)	0.0174 (17)	−0.028 (2)
N1A	0.0386 (12)	0.0714 (16)	0.0622 (15)	0.0011 (12)	0.0000 (11)	−0.0223 (13)
O1A	0.0450 (11)	0.0824 (15)	0.0615 (13)	−0.0052 (10)	−0.0012 (9)	−0.0166 (11)
O2A	0.0605 (13)	0.0747 (14)	0.0665 (14)	0.0030 (11)	0.0150 (10)	−0.0227 (12)
O3A	0.0395 (10)	0.0911 (15)	0.0756 (14)	−0.0023 (11)	0.0092 (10)	−0.0285 (12)
C1B	0.0615 (19)	0.101 (3)	0.059 (2)	−0.0030 (18)	0.0166 (16)	−0.0048 (18)
C2B	0.0498 (16)	0.0672 (18)	0.0488 (17)	−0.0035 (14)	0.0040 (13)	−0.0018 (14)
C3B	0.064 (2)	0.102 (3)	0.0503 (19)	−0.0097 (19)	−0.0019 (15)	−0.0053 (18)
C4B	0.0501 (18)	0.105 (3)	0.075 (2)	−0.0117 (18)	−0.0063 (17)	−0.002 (2)
C5B	0.0491 (18)	0.093 (3)	0.080 (3)	−0.0057 (17)	0.0119 (17)	0.000 (2)
C6B	0.0597 (18)	0.0678 (19)	0.0516 (18)	0.0000 (15)	0.0127 (15)	−0.0013 (15)
C7B	0.0470 (15)	0.0499 (15)	0.0460 (16)	−0.0019 (12)	0.0005 (12)	0.0006 (12)
C8B	0.0542 (17)	0.0606 (19)	0.060 (2)	0.0039 (15)	−0.0015 (15)	−0.0007 (16)
C9B	0.133 (4)	0.078 (3)	0.157 (4)	−0.046 (3)	−0.031 (3)	0.035 (3)
N1B	0.0566 (14)	0.0528 (14)	0.0468 (14)	−0.0014 (11)	−0.0043 (11)	0.0066 (11)
O1B	0.0638 (13)	0.0631 (13)	0.0490 (12)	0.0123 (10)	0.0050 (9)	0.0096 (10)
O2B	0.0765 (15)	0.0873 (17)	0.0722 (15)	−0.0163 (13)	−0.0246 (13)	−0.0017 (13)
O3B	0.0747 (15)	0.0585 (13)	0.0865 (16)	−0.0123 (11)	−0.0162 (12)	0.0143 (12)
C1C	0.098 (3)	0.085 (3)	0.142 (4)	−0.010 (2)	0.040 (3)	0.004 (3)
C2C	0.086 (3)	0.079 (2)	0.091 (3)	−0.004 (2)	0.024 (2)	0.004 (2)
C3C	0.087 (3)	0.129 (4)	0.109 (3)	0.042 (3)	−0.001 (3)	0.036 (3)
C4C	0.085 (3)	0.170 (5)	0.135 (4)	0.029 (3)	−0.006 (3)	0.061 (4)
C5C	0.063 (2)	0.152 (4)	0.127 (4)	−0.007 (3)	−0.004 (2)	0.031 (3)
C6C	0.063 (2)	0.089 (3)	0.087 (3)	0.0022 (19)	0.0027 (19)	0.016 (2)
C9C	0.109 (3)	0.127 (3)	0.057 (2)	0.030 (3)	−0.008 (2)	−0.035 (2)
C7C	0.073 (2)	0.0644 (19)	0.0556 (19)	0.0068 (17)	−0.0026 (16)	−0.0058 (16)
C8C	0.0504 (17)	0.0554 (17)	0.0577 (19)	0.0015 (14)	0.0013 (14)	−0.0084 (15)
N1C	0.0570 (14)	0.0695 (16)	0.0505 (15)	0.0180 (12)	−0.0002 (12)	−0.0072 (12)
O1C	0.0628 (13)	0.0682 (13)	0.0579 (13)	0.0055 (10)	0.0108 (10)	−0.0100 (11)
O2C	0.0620 (13)	0.0795 (14)	0.0607 (14)	0.0194 (12)	−0.0091 (10)	−0.0087 (11)
O3C	0.0716 (14)	0.0880 (16)	0.0612 (14)	0.0252 (12)	−0.0039 (11)	−0.0230 (12)

### Geometric parameters (Å, º)

**Table:**

C1A—C2A	1.429 (6)	C6B—C7B	1.385 (4)
C1A—H1A1	0.9600	C6B—H6B	0.9300
C1A—H1A2	0.9600	C7B—N1B	1.432 (3)
C1A—H1A3	0.9600	C8B—O2B	1.208 (3)
C2A—C7A	1.359 (5)	C8B—O3B	1.335 (3)
C2A—C3A	1.416 (5)	C8B—N1B	1.350 (4)
C3A—C4A	1.330 (6)	C9B—O3B	1.445 (4)
C3A—H3A	0.9300	C9B—H9B1	0.9600
C4A—C5A	1.398 (7)	C9B—H9B2	0.9600
C4A—H4A	0.9300	C9B—H9B3	0.9600
C5A—C6A	1.357 (6)	N1B—O1B	1.407 (3)
C5A—H5A	0.9300	O1B—H1B	0.8200
C6A—C7A	1.429 (5)	C1C—C2C	1.464 (5)
C6A—H6A	0.9300	C1C—H1C1	0.9600
C7A—N1A	1.425 (4)	C1C—H1C2	0.9600
C8A—O2A	1.212 (3)	C1C—H1C3	0.9600
C8A—O3A	1.332 (3)	C2C—C7C	1.351 (5)
C8A—N1A	1.350 (3)	C2C—C3C	1.423 (5)
C9A—O3A	1.445 (3)	C3C—C4C	1.333 (6)
C9A—H9A1	0.9600	C3C—H3C	0.9300
C9A—H9A2	0.9600	C4C—C5C	1.389 (6)
C9A—H9A3	0.9600	C4C—H4C	0.9300
N1A—O1A	1.410 (3)	C5C—C6C	1.337 (5)
O1A—H1A	0.8200	C5C—H5C	0.9300
C1B—C2B	1.511 (4)	C6C—C7C	1.428 (5)
C1B—H1B1	0.9600	C6C—H6C	0.9300
C1B—H1B2	0.9600	C9C—O3C	1.438 (4)
C1B—H1B3	0.9600	C9C—H9C1	0.9600
C2B—C7B	1.389 (4)	C9C—H9C2	0.9600
C2B—C3B	1.390 (4)	C9C—H9C3	0.9600
C3B—C4B	1.374 (4)	C7C—N1C	1.432 (4)
C3B—H3B	0.9300	C8C—O2C	1.212 (3)
C4B—C5B	1.369 (5)	C8C—N1C	1.337 (4)
C4B—H4B	0.9300	C8C—O3C	1.339 (3)
C5B—C6B	1.377 (4)	N1C—O1C	1.403 (3)
C5B—H5B	0.9300	O1C—H1C	0.8200
			
C2A—C1A—H1A1	109.5	C5B—C6B—H6B	120.2
C2A—C1A—H1A2	109.5	C7B—C6B—H6B	120.2
H1A1—C1A—H1A2	109.5	C6B—C7B—C2B	121.5 (3)
C2A—C1A—H1A3	109.5	C6B—C7B—N1B	118.8 (3)
H1A1—C1A—H1A3	109.5	C2B—C7B—N1B	119.7 (2)
H1A2—C1A—H1A3	109.5	O2B—C8B—O3B	124.0 (3)
C7A—C2A—C3A	119.4 (4)	O2B—C8B—N1B	125.3 (3)
C7A—C2A—C1A	121.9 (4)	O3B—C8B—N1B	110.6 (3)
C3A—C2A—C1A	118.7 (4)	O3B—C9B—H9B1	109.5
C4A—C3A—C2A	117.7 (5)	O3B—C9B—H9B2	109.5
C4A—C3A—H3A	121.1	H9B1—C9B—H9B2	109.5
C2A—C3A—H3A	121.1	O3B—C9B—H9B3	109.5
C3A—C4A—C5A	125.5 (5)	H9B1—C9B—H9B3	109.5
C3A—C4A—H4A	117.3	H9B2—C9B—H9B3	109.5
C5A—C4A—H4A	117.3	C8B—N1B—O1B	113.4 (2)
C6A—C5A—C4A	116.5 (5)	C8B—N1B—C7B	125.2 (2)
C6A—C5A—H5A	121.8	O1B—N1B—C7B	115.3 (2)
C4A—C5A—H5A	121.8	N1B—O1B—H1B	109.5
C5A—C6A—C7A	120.3 (5)	C8B—O3B—C9B	115.9 (3)
C5A—C6A—H6A	119.8	C2C—C1C—H1C1	109.5
C7A—C6A—H6A	119.8	C2C—C1C—H1C2	109.5
C2A—C7A—N1A	122.9 (3)	H1C1—C1C—H1C2	109.5
C2A—C7A—C6A	120.5 (3)	C2C—C1C—H1C3	109.5
N1A—C7A—C6A	116.5 (3)	H1C1—C1C—H1C3	109.5
O2A—C8A—O3A	124.5 (2)	H1C2—C1C—H1C3	109.5
O2A—C8A—N1A	124.4 (3)	C7C—C2C—C3C	116.6 (4)
O3A—C8A—N1A	111.0 (2)	C7C—C2C—C1C	121.7 (4)
O3A—C9A—H9A1	109.5	C3C—C2C—C1C	121.6 (4)
O3A—C9A—H9A2	109.5	C4C—C3C—C2C	121.1 (4)
H9A1—C9A—H9A2	109.5	C4C—C3C—H3C	119.4
O3A—C9A—H9A3	109.5	C2C—C3C—H3C	119.4
H9A1—C9A—H9A3	109.5	C3C—C4C—C5C	121.6 (4)
H9A2—C9A—H9A3	109.5	C3C—C4C—H4C	119.2
C8A—N1A—O1A	114.0 (2)	C5C—C4C—H4C	119.2
C8A—N1A—C7A	126.8 (2)	C6C—C5C—C4C	119.4 (4)
O1A—N1A—C7A	114.3 (2)	C6C—C5C—H5C	120.3
N1A—O1A—H1A	109.5	C4C—C5C—H5C	120.3
C8A—O3A—C9A	115.3 (2)	C5C—C6C—C7C	119.3 (4)
C2B—C1B—H1B1	109.5	C5C—C6C—H6C	120.4
C2B—C1B—H1B2	109.5	C7C—C6C—H6C	120.4
H1B1—C1B—H1B2	109.5	O3C—C9C—H9C1	109.5
C2B—C1B—H1B3	109.5	O3C—C9C—H9C2	109.5
H1B1—C1B—H1B3	109.5	H9C1—C9C—H9C2	109.5
H1B2—C1B—H1B3	109.5	O3C—C9C—H9C3	109.5
C7B—C2B—C3B	117.1 (3)	H9C1—C9C—H9C3	109.5
C7B—C2B—C1B	121.8 (3)	H9C2—C9C—H9C3	109.5
C3B—C2B—C1B	121.1 (3)	C2C—C7C—C6C	121.9 (3)
C4B—C3B—C2B	121.6 (3)	C2C—C7C—N1C	120.9 (3)
C4B—C3B—H3B	119.2	C6C—C7C—N1C	117.1 (3)
C2B—C3B—H3B	119.2	O2C—C8C—N1C	125.3 (3)
C5B—C4B—C3B	120.1 (3)	O2C—C8C—O3C	124.2 (3)
C5B—C4B—H4B	119.9	N1C—C8C—O3C	110.5 (3)
C3B—C4B—H4B	119.9	C8C—N1C—O1C	114.8 (2)
C4B—C5B—C6B	120.0 (3)	C8C—N1C—C7C	127.5 (2)
C4B—C5B—H5B	120.0	O1C—N1C—C7C	117.7 (2)
C6B—C5B—H5B	120.0	N1C—O1C—H1C	109.5
C5B—C6B—C7B	119.5 (3)	C8C—O3C—C9C	115.1 (3)
			
C7A—C2A—C3A—C4A	1.2 (6)	O2B—C8B—N1B—O1B	−13.3 (4)
C1A—C2A—C3A—C4A	179.4 (4)	O3B—C8B—N1B—O1B	169.4 (2)
C2A—C3A—C4A—C5A	−2.2 (7)	O2B—C8B—N1B—C7B	−164.4 (3)
C3A—C4A—C5A—C6A	3.4 (7)	O3B—C8B—N1B—C7B	18.4 (4)
C4A—C5A—C6A—C7A	−3.4 (6)	C6B—C7B—N1B—C8B	101.2 (3)
C3A—C2A—C7A—N1A	−178.1 (3)	C2B—C7B—N1B—C8B	−79.5 (4)
C1A—C2A—C7A—N1A	3.8 (5)	C6B—C7B—N1B—O1B	−49.4 (3)
C3A—C2A—C7A—C6A	−1.5 (5)	C2B—C7B—N1B—O1B	129.9 (3)
C1A—C2A—C7A—C6A	−179.6 (4)	O2B—C8B—O3B—C9B	−3.8 (5)
C5A—C6A—C7A—C2A	2.8 (5)	N1B—C8B—O3B—C9B	173.5 (3)
C5A—C6A—C7A—N1A	179.5 (3)	C7C—C2C—C3C—C4C	0.1 (7)
O2A—C8A—N1A—O1A	−9.2 (4)	C1C—C2C—C3C—C4C	179.9 (5)
O3A—C8A—N1A—O1A	173.4 (2)	C2C—C3C—C4C—C5C	−1.1 (9)
O2A—C8A—N1A—C7A	−162.9 (3)	C3C—C4C—C5C—C6C	0.4 (9)
O3A—C8A—N1A—C7A	19.7 (4)	C4C—C5C—C6C—C7C	1.2 (7)
C2A—C7A—N1A—C8A	−117.7 (4)	C3C—C2C—C7C—C6C	1.5 (5)
C6A—C7A—N1A—C8A	65.6 (4)	C1C—C2C—C7C—C6C	−178.3 (4)
C2A—C7A—N1A—O1A	88.7 (4)	C3C—C2C—C7C—N1C	178.9 (3)
C6A—C7A—N1A—O1A	−88.0 (3)	C1C—C2C—C7C—N1C	−1.0 (5)
O2A—C8A—O3A—C9A	−0.5 (4)	C5C—C6C—C7C—C2C	−2.2 (6)
N1A—C8A—O3A—C9A	177.0 (3)	C5C—C6C—C7C—N1C	−179.6 (4)
C7B—C2B—C3B—C4B	1.2 (5)	O2C—C8C—N1C—O1C	−4.7 (4)
C1B—C2B—C3B—C4B	−177.8 (3)	O3C—C8C—N1C—O1C	174.7 (2)
C2B—C3B—C4B—C5B	0.8 (6)	O2C—C8C—N1C—C7C	176.6 (3)
C3B—C4B—C5B—C6B	−1.5 (6)	O3C—C8C—N1C—C7C	−4.0 (4)
C4B—C5B—C6B—C7B	0.2 (5)	C2C—C7C—N1C—C8C	111.8 (4)
C5B—C6B—C7B—C2B	1.9 (4)	C6C—C7C—N1C—C8C	−70.7 (4)
C5B—C6B—C7B—N1B	−178.8 (3)	C2C—C7C—N1C—O1C	−66.8 (4)
C3B—C2B—C7B—C6B	−2.6 (4)	C6C—C7C—N1C—O1C	110.6 (3)
C1B—C2B—C7B—C6B	176.4 (3)	O2C—C8C—O3C—C9C	−6.0 (5)
C3B—C2B—C7B—N1B	178.2 (3)	N1C—C8C—O3C—C9C	174.6 (3)
C1B—C2B—C7B—N1B	−2.9 (4)		

### Hydrogen-bond geometry (Å, º)

**Table:**

*D*—H···*A*	*D*—H	H···*A*	*D*···*A*	*D*—H···*A*
O1*A*—H1*A*···O2*B*	0.82	1.94	2.719 (3)	157
O1*B*—H1*B*···O2*C*	0.82	1.94	2.716 (3)	157
O1*C*—H1*C*···O2*A*	0.82	1.99	2.757 (3)	156

## References

[bb1] Bernstein, J., Davis, R. E., Shimoni, L. & Chang, N.-L. (1995). *Angew. Chem. Int. Ed. Engl.* **34**, 1555–1573.

[bb2] Farrugia, L. J. (2012). *J. Appl. Cryst.* **45**, 849–854.

[bb3] Higashi, T. (1995). *ABSCOR* Rigaku Corporation, Tokyo, Japan.

[bb4] Hou, C.-Q., Li, Z.-N. & Liu, C.-L. (2002). *Pesticides*, **41**, 41–43.

[bb5] Mercader, J. V., Agullo, C., Abad-Somovilla, A. & Abad-Fuentes, A. (2011). *Org. Biomol. Chem.* **9**, 1443–1453. 10.1039/c0ob00686f 21225057

[bb6] Rigaku (2006). PROCESS_AUTO. Rigaku Corporation, Tokyo, Japan.

[bb7] Rigaku (2007). *CrystalStructure.* Rigaku Americas, The Woodlands, Texas, USA.

[bb8] Sheldrick, G. M. (2008). *Acta Cryst.* A**64**, 112–122. 10.1107/S0108767307043930 18156677

[bb9] Tao, X.-J., Luo, L.-M., Huang, C.-Q. & Xiong, L.-L. (2009). *Agrochem. Res. Appl.* **41**, 41–43.

[bb10] Yang, L.-J. & Bai, Y.-L. (2012). *Mod. Agrochem.* **11**, 46–50.

